# The Online Dissemination of Nature–Health Concepts: Lessons from Sentiment Analysis of Social Media Relating to “Nature-Deficit Disorder”

**DOI:** 10.3390/ijerph13010142

**Published:** 2016-01-19

**Authors:** Marco Palomino, Tim Taylor, Ayse Göker, John Isaacs, Sara Warber

**Affiliations:** 1 School of Computing and Digital Media, Robert Gordon University, Aberdeen, Scotland AB10 7GE, UK; a.s.goker@rgu.ac.uk (A.G.); j.p.isaacs@rgu.ac.uk (J.I.); 2 European Centre for Environment and Human Health, University of Exeter Medical School, Truro, Cornwall TR1 3HD, UK; timothy.j.taylor@exeter.ac.uk (T.T.); swarber@umich.edu (S.W.); 3 Department of Family Medicine, University of Michigan Medical School, Ann Arbor, MI 48104-1213, USA

**Keywords:** nature-deficit disorder, sentiment analysis, Twitter, big data, nature–health

## Abstract

Evidence continues to grow supporting the idea that restorative environments, green exercise, and nature-based activities positively impact human health. *Nature-deficit disorder*, a journalistic term proposed to describe the ill effects of people’s alienation from nature, is not yet formally recognized as a medical diagnosis. However, over the past decade, the phrase has been enthusiastically taken up by some segments of the lay public. Social media, such as *Twitter*, with its opportunities to gather “big data” related to public opinions, offers a medium for exploring the discourse and dissemination around *nature-deficit disorder* and other nature–health concepts. In this paper, we report our experience of collecting more than 175,000 tweets, applying *sentiment analysis* to measure positive, neutral or negative feelings, and preliminarily mapping the impact on dissemination. Sentiment analysis is currently used to investigate the repercussions of events in social networks, scrutinize opinions about products and services, and understand various aspects of the communication in Web-based communities. Based on a comparison of nature-deficit-disorder “hashtags” and more generic nature hashtags, we make recommendations for the better dissemination of public health messages through changes to the framing of messages. We show the potential of Twitter to aid in better understanding the impact of the natural environment on human health and wellbeing.

## 1. Introduction

Research in public health [1], environmental psychology [2], landscape architecture [3] and other disciplines continues to accumulate supporting the idea that nearby natural environments [4], green exercise [5,6], and nature-based activities [7] positively impact human health and wellbeing. At the same time, the urbanized, media-based culture is increasingly linked to a more sedentary lifestyle and poorer health [8,9] and is suspected of decreasing both time in nature and connection to nature [10]. For example, a growing corpus of literature suggests an explicit correlation between the lack of outdoor activity and obesity-related ailments like diabetes [11].

*Nature-deficit disorder* (*NDD*) is a term proposed by journalist Richard Louv in 2005 to describe these ill effects of people’s separation from nature. Louv argues that the human cost of “alienation from nature” is measured in “diminished use of the senses, attention difficulties and higher rates of physical and emotional illnesses” [12]. *NDD* has resonance with lay audiences, especially parents, educators, and environmental non-profit organizations. In the UK, for instance, concern over *NDD* motivated the *National Trust*, whose mission is to preserve places of historic interest and natural beauty, to propose programs to ensure that every child has the chance to develop a personal connection with the natural world [13]. A recent mixed methods study suggests that young people’s connection to nature and their holistic wellbeing is increased after a wilderness camp experience, thus addressing *NDD* [14].

In contrast, *NDD* is not generally recognized as a medical diagnosis or a public health issue. To demonstrate the differential between public discourse about *NDD* and the health-related discourse, a Google search of the World Wide Web produces more than 800,000 hits for *NDD*, while a search of PubMed produces only one [15]. However, illness and diagnosis is a social construction [16] so an exploration of social media may offer new ways of investigating public opinion on this topic. The growth of the Web 2.0 and the rising popularity of social platforms such as *Twitter* and *Facebook* have provided opportunities for people to express their opinions publicly more often than ever before.

The use of Twitter for both research purposes and as part of specific interventions is growing in the field of public health, especially in the last few years [17]. Twitter has been used to track emerging diseases [18]; disseminate health-related messages [19]; and understand the public’s views, knowledge, attitudes, beliefs, and behaviors [20,21]. Information-mining of social media has led to the development of a new area of research, known as *sentiment analysis*, the objective of which is to translate opinions and expressions of human emotion into data that can be quantified and categorized to determine the attitude towards particular topics, services or products [22]. Sentiment analysis is currently being used in the business and social domain [23], including public health [24]. Commercially speaking, online opinions are seen as an invaluable source of information—indeed, some organizations employ people dedicated to read posts on social websites and extract insight into what is being said about their products, services and competitors [25].

In terms of this study, it has been shown that general attitude and commentary about the human relationship with nature and interests in outdoor activities have lately become a popular topic of debate and conversation within social media platforms [26]; especially, in *social awareness streams* (SAS) [27] like those found in Facebook and Twitter, where people post content that is available immediately, publicly or semi-publicly.

The goal of this article, then, is to utilize Twitter to explore the online dissemination and sentiment associated with the concept of *NDD* and other nature–health related concepts. Our work aims to build a dataset that will enable in-depth qualitative analysis of tweets linked to *NDD* published by the general public. Mining the “big data” of Twitter to study the social construction of nature and health, *NDD*, or other important public health messages requires new tools and techniques, which we intend to both explicate and critique. To the best of our knowledge, no previous study of this kind on this subject has been carried out, though this has potential to help to understand the relevance of *NDD* and related nature–health concepts in the community—and their online dissemination—and in turn suggest recommendations for policy makers.

## 2. Experimental Section

To carry out our work, we began by choosing *Twitter* [28], as the platform where our research was undertaken. Twitter is a good starting point for social media analysis, because its users tend to share their opinions openly with the general public, as opposed to Facebook, where interactions are frequently private or semi-private—restricted to designated contacts or “friends”.

### 2.1. Twitter

Twitter is a service that helps people create and share ideas and information instantly. It offers an easy way to follow trends, stories, and breaking news making headlines around the world, and it also provides a mechanism to stay in touch with other people, businesses and social causes. Precisely, Twitter is an information network made up of 140-character messages called *tweets* [29]. Tweets may contain links to other websites, articles, photos and videos.

In the context of Twitter, a “hashtag” is any word or phrase immediately preceded by a hash sign (#) [30]. Hashtags are used to identify posts on a specific topic—for instance, when a user clicks on a hashtag, she will see other tweets containing the same keyword or topic. Hashtags can occur anywhere in a tweet—at the beginning, middle, or end. Hashtags that become very popular are often considered *trending topics* [30].

A tweet that a user forwards to all of her “followers”, or designated contacts within Twitter, is known as a “retweet”, (*RT*). Retweets are often used to pass along news or other valuable discoveries on Twitter. It should be observed that retweets always retain original attribution.

The method that we pursued to retrieve relevant tweets is described below.

### 2.2. NDD Hashtags

Although a number of online communities and portals to encourage and support people and organizations working nationally and internationally to connect children with nature have been created lately—for example, the *Children & Nature Network* (childrenandnature.org)—we did not focus our investigation on specific communities where *NDD* advocates are likely to gather up. Rather than monitoring particular websites, we aimed to capture the overall sentiment expressed in Twitter by anyone referring to *NDD*. Since the number of tweets published on a daily basis is too high (on a typical day, more than 500 million tweets are published; an average of 5700 tweets per second [31]), and most of them are unlikely to allude to *NDD*, we restricted our study to a collection of hashtags suggested by one of us (SW) and vetted among scholars with knowledge and involvement in the field of nature and human health. Such hashtags were selected to include phrases directly associated with *NDD*—e.g., #naturedeficitdisorder—phrases that were conceptually related to *NDD*—e.g., #playoutside—phrases about general nature and health—e.g., #natureheals—and also generic nature terms—e.g., #outdoors. Table 1 shows the whole list of hashtags involved in this study and the total number of tweets retrieved for each particular hashtag over the 2-month period that we investigated—between 1 July 2014 and 31 August 2014. The only phrase included in our study that is not a hashtag is “nature deficit disorder”, which appears in the *NDD* section of Table 1. Note that some of the hashtags in Table 1 do not refer to *NDD* explicitly, but to concepts and phrases connected to *NDD*. For instance, “Last Child in the Woods” is the title of the book where Louv introduced the term *NDD*; “Park Prescriptions” is a movement to create a healthier population by strengthening the links between the healthcare system and public lands across the US; and “The Nature Principle” is the title of another book by Louv—yet, no tweets including the hashtag #thenatureprinciple were posted while our research took place. Indeed, only one tweet including the #thenatureprinciple was posted in 2014 and this was on 7 September 2014—a week after our retrieval of tweets had ended.

**Table 1:** Hashtags and phrases.

**NDD Hashtags/Phrases**	**Tweets Retrieved**
#lastchildinthewoods	14
#leavenochildinside	5
#naturedeficitdisorder	57
#nochildleftindoors	3
#thenatureprinciple	0
#vitamin N	54
nature deficit disorder	401
**NDD-Related Hashtags**	**Tweets Retrieved**
#getoutside	6333
#goplay	1739
#outdoorfamilies	1098
#playoutside	748
#qualityfamilytimeinnature	0
**Nature–Health Hashtags**	**Tweets Retrieved**
#naturecures	11
#natureheals	514
#parkprescriptions	37
**Generic-Nature Hashtags**	**Tweets Retrieved**
#green	32,722
#outdoors	20,586
#wildlife	112,172

### 2.3. Twitter’s API

We retrieved the tweets for our study using the open source, Twitter *application programming interface* (API) library *Twitter4J* [32]. At the time we performed our study, we were only able to retrieve tweets less than 7 days old, and we could only place 180 requests for tweets every 15 min. To collect such tweets, we issued a separate request for each hashtag via the *Twitter Search API* [31]—in other words, we performed a separate retrieval process for each hashtag in our study.

The Twitter Search API [33] behaves similarly to the search feature available in Twitter. However, the Twitter Search API focuses on relevance, as opposed to completeness [33], which means that some tweets and users may be missing from the search results. In any case, at the time of our study, the Search API represented the most convenient way to retrieve tweets for our purposes.

To accumulate as many tweets as possible—ideally all the tweets published for each of the hashtags chosen—we issued our queries twice a day: at 9:00 a.m. and 5:00 p.m. Certain tweets corresponding to particular hashtags were captured in the morning and once again in the evening—when the number of tweets published for those hashtags on a single day was smaller than the number of tweets that we were allowed to retrieve. For the same reason, some of the tweets captured in the evening were recaptured the following morning too.

After retrieving tweets, we removed duplicates to ensure that every tweet in our study was considered only once. The only case in which a tweet was considered more than once was when the tweet referred to two or more hashtags or phrases involved in the study. For example, the following tweet contains the hashtag #naturedeficitdisorder and the phrase “Nature Deficit Disorder”. Therefore, we processed it twice: first for the hashtag #naturedeficitdisorder and then for the phrase “Nature Deficit Disorder”.

Nature Deficit Disorder and climate change widen the debate for a future worth having #naturedeficitdisorder https://t.co/GEavvJ1ZFn.

### 2.4. Sentiment Analysis

Sentiment analysis—or *opinion mining*—is concerned with the use of natural language processing and computational linguistics to identify and extract subjective information in text materials, such as tweets. A wide range of human moods can be discovered through sentiment analysis, but a major focus has been identifying the *polarity* of a given text [34]—*i.e.*, to automatically recognize if a text is *positive*, *negative* or *neutral*.

One of the earliest studies on tweet polarity was done by Go *et al.* [35], who conducted a classification analysis of tweets in English using *emoticons*—for instance, “:)” and “:(“—as markers of positive and negative tweets. Read [36] and Pak and Paroubek [37] also worked on tweet polarity, combining the detection of specific emoticons with other methods.

Mixing supervised learning with the recognition of sentiment-bearing words stored in sentiment dictionaries [38,39] has been considered too, and that is the approach that we followed; however, instead of developing our own implementation, we made use of *AlchemyAPI* [40]. By leveraging the infrastructure of a specialist on sentiment analysis, we automated the process of identifying the polarity of tweets. A description of AlchemyAPI and its API is offered below.

### 2.5. AlchemyAPI

AlchemyAPI (AlchemyAPI™ is an IBM Company, part of Watson Developer Cloud.) is a text mining platform providing semantic analysis capabilities. It is piece of software used over 3 billion times globally per month, on average, enabling academics and commercial firms to perform social media monitoring and sentiment analysis [41]. In March 2015, IBM acquired AlchemyAPI to accelerate its development of cognitive computing applications [42].

As independent research has demonstrated, AlchemyAPI’s sentiment analysis is uniquely positioned. Meehan *et al.* showed that AlchemyAPI’s sentiment analysis achieved an 86% accuracy after manual testing carried out on a corpus of 5370 tweets employed by an intelligent recommendation system for tourism [43]. Rizzo and Troncy [44] and Saif *et al.* [45] have also validated the performance of AlchemyAPI on a number of datasets and in different contexts: Rizzo and Troncy found out that AlchemyAPI is better at extracting named entities and categorizing them than other semantic entity extractors [44]—such as, *Zemanta* [46], *OpenCalais* [47], *Extractiv* [48] and *DBpedia Spotlight* [49]. Similarly, Saif *et al.* favored AlchemyAPI over OpenCalais and Zemanta for the task of concept extraction, due to its better coverage [45].

Based on the evaluations stated in previous research, we believe that AlchemyAPI is a suitable choice to support our work.

### 2.6. Determination of Sentiment in Tweets

Every time the AlchemyAPI’s sentiment analysis API is invoked for a tweet, it returns the following information:

(1) The sentiment *polarity*, which can be *positive*—“this car is great”—*negative*—“this car is overpriced”—or *neutral*—“this car is red”.

(2) The sentiment *strength*, which is a real number value between −1 and 1 that expresses how negative or positive the sentiment is—zero means that the sentiment is neutral, negative values refer to negative sentiment and positive values refer to positive sentiment.

(3) A Boolean value to indicate whether the sentiment is *mixed*—*i.e.*, both positive and negative. Note that the same tweet or statement in general can be negative about a person or product and positive about something else.

For illustration purposes, Table 2 displays a random sample of tweets taken from our study together with their corresponding polarity, score and mixed values, as retrieved from AlchemyAPI. For research purposes, AlchemyAPI offers its services for free. However, it only allows 1000 transactions daily—determining the polarity of one tweet represents a single transaction. From the 1000 transactions that we were allowed daily, we kept 5 for testing, and then we submitted a daily batch of requests for the polarity of 995 different tweets. Although the testing was only indispensable for the first few days, when our experience in using AlchemyAPI was limited, we maintained the same approach for the rest of the experiment, as this preserved a constant number of transactions daily without exceeding the daily limit.

It took 178 consecutive days—*i.e.*, nearly 6 months—to determine the polarity of the entire collection of tweets in our experiment (176,494). We could have completed the processing earlier, by exclusively requesting the polarity of original tweets and assigning the same polarity to all the retweets—this would have saved us 80 days of processing, approximately. However, retweets frequently remove the last few characters of an original tweet, because there is not enough space to keep the whole content posted initially—recall that tweets cannot be longer than 140 characters (occasionally, retweets also contain additional content—for example, a small comment that constitutes what is known as a *quote tweet*). Since the polarity scores supplied by AlchemyAPI are based on the whole content, and this may change slightly between a tweet and a retweet, we decided to process the retweets separately.

**Table 2:** Sample tweets processed by AlchemyAPI.

**NDD Hashtags/Phrases**				
**Tweet**	**Polarity**		**Score**	**Mixed**
Best day on the water is one spent with my boys! #familyfunday #nochildleftindoors #boating #sandiego… http://t.co/XScgRxyNMx	positive		0.466531	No
So you’re camping but I can clearly see that you’re watching the big screen tv in your RV. #AMERICA #naturedeficitdisorder	negative		−0.643313	Yes
#NatureDeficitDisorder predicted to rise as kids herded back into classrooms for the next 12 months. Yeah Yeah for #technology	negative		−0.720397	No
**NDD-Related Hashtags**				
**Tweet**	**Polarity**		**Score**	**Mixed**
The best thing that could happen to this country would be social media being shut down... #getoffyourphone and #getoutside	neutral		0	
It's time we gave our children permission to get outside and get dirty|Helen Meech http://t.co/nYFW8CkxWI via @guardian #outdoorfamilies	neutral		0	
“@TechnologicFact: Humanity has spent a collective 200,000 years playing Angry Birds” this fact makes me Angry Man #playoutside	negative		−0.62595	Yes
Go play outside! Outdoor time promotes physical activity in youth http://t.co/UPvyJQxxZo #playoutside	neutral		0	
**Nature–Health Hashtags**				
**Tweet**	**Polarity**	**Score**		**Mixed**
RT @IntBirdRescue: “I am well again, I came to life in the cool winds and crystal waters of the mountains.” ~John Muir #NatureHeals	positive		0.578088	No
Nothing cleanses your thoughts like a hike in the rain. #feelingblessed #naturecures #meditate http://t.co/wlbrmsCtdN	positive		0.0505909	Yes
**NDD-Related Hashtags**				
**Tweet**	**Polarity**		**Score**	**Mixed**
#Camping is a great way to bond with the kids and to get them #outdoors and exercising http://t.co/4O9cYqIDVV	positive		0.903222	No
-- Although I don’t really like the smell of rain; it reminds me of my younger years “Playing out” after the rain had stopped!! #Outdoors	positive		0.327455	...

Table 3 displays an example of a tweet whose polarity score is marginally greater than the score of its corresponding retweet. This is caused by the word effective, which has a positive connotation, appearing at the end of the original tweet, but being partly removed from the retweet due to lack of space. We realize that a retweet is meant to pass along the same sentiment expressed in the original tweet, but we opted to account for marginal changes in polarity caused by minor changes in content.

**Table 3:** Tweet *vs.* retweet polarity.

Tweet	Polarity	Score
I love how social media can be so encouraging to #getactive and #getoutside. Seemingly paradoxical, but wow, it's effective. #FSUWEPO	positive	0.843067
RT @max_mckaig: I love how social media can be so encouraging to #getactive and #getoutside. Seemingly paradoxical, but wow, it's effecti...	positive	0.71574

### 2.7. Exclusions

Certain hashtags considered in our study, such as #outdoors, are not only of interest to *NDD* advocates, but are also used, extensively, by adult-content communities to share references to nudity and sexuality that are not relevant to *NDD*. Thus, we removed 11,244 tweets from the #outdoors collection that contained hashtags such as #nudist and #adult. It should be observed that the total number of tweets retrieved belonging to #outdoors was 31,830; yet, the number shown in Table 1 (20,586) refers to the tweets retained after removing adult content. Also, it is worth noting that no adult content was found in the tweets corresponding to other hashtags, apart from #outdoors.

Even though we aimed to capture tweets written in English, exclusively, a number of tweets in non-English languages were retrieved too. This is largely because some users typed their tweets in other languages, but “labeled” them with the hashtags chosen for our study.

Table 4 displays some examples of non-English tweets included in our collection—we have highlighted in bold font the hashtags whose presence caused the retrieval of these tweets. While we acknowledge the importance of tweets in non-English languages to analyze the worldwide recognition of *NDD*, we opted to discard them from our evaluation, because we did not have enough resources to translate them into English or process them in their respective languages.

**Table 4:** Tweets in non-English languages.

Tweet	Translation
Dieci consigli per vivere #green e per fare della tua città un posto piacevole./#ambiente #natura|http://t.co/wH4oPRWLjI	Ten tips for living #green and make your city a pleasant place./#ambiente #natura|http://t.co/wH4oPRWLjI
Les gens doivent se faire chier... Pourquoi venir chatter alors qu’il fait beau dehors et que ce sont les vacances ? #getoutside #work	People must be bored as hell... Why go on chat when the weather is good and it's the holidays? #getoutside #work
Un cargador enchufado sin uso, en un año consume la electricidad de 5 casas. Desconéctalo si no lo utilizas, ahorrarás mucho #green	An unused charger plugged in for a year consumes the electricity of 5 houses. If you do not use it, disconnect it, you will save a great deal #green

Another reason why we discarded some tweets from our evaluation is that AlchemyAPI was unable to identify any intelligible text within them. This might be caused by tweets originally written in languages whose alphabets are not Latin—for example, languages using the Cyrillic or Japanese alphabet. As a result, Twitter4J misses the original encoding and we end up collecting a series of punctuation marks accompanied by some hashtags and links to other resources.

Table 5 exhibits some examples of tweets made up of a combination of punctuation marks, hashtags and links, but no comprehensible text. Since a significant part of the tweet is unintelligible, AlchemyAPI could not determine its polarity. In total, we removed 6538 tweets for being unintelligible or written in non-English languages.

**Table 5:** Tweets without recognizable text.

Tweet	Presumed Location
?? 2014.07.06 ?? ????????????? ????????????????????????????? #??? #green #?? #nature #???? @ ?????? http://t.co/ETSbxjghsW	Sendai, Miyagi, Japan
??????? «??????????? ????» ????????? ??? ????????? ???????? 1200 ???? ????? http://t.co/UFPE9KHsap #wildlife #????????	Belarus

Table 6 shows general statistics about our collection of tweets, including the number of tweets attached to one or more hashtags or phrases, the number of users and the number of retweets involved. Apart from #green, #outdoors and #wildlife, our retrieval strategy sufficed to collect all the available tweets for each hashtag in the 2-month period studied—recall that Twitter4J only allowed us to retrieve tweets less than 7 days old, and we could only place 180 requests for tweets every 15 min. In addition, we only attempted two retrievals per hashtag per day.

**Table 6:** General statistics.

Twitter Hashtag/Phrases	Tweets Retrieved
Number of tweets retrieved	187,738
Tweets removed due to adult content	11,244
Number of tweets considered in the study	176,494
**Unique (non-duplicate) tweets considered**	**176,219**
Tweets containing 1 hashtag/phrase	174,690
Tweets containing 2 hashtags/phrases	1788
Tweets containing 3 or more hashtags/phrases	16
Unique users	75,105
Users removed due to adult content	620
**Unique users considered for the study**	**74,485**
Number of retweets	79,323
Retweets removed due to adult content	789
**Number of retweets considered in the study**	**78,531**

## 3. Results and Discussion

### 3.1. Sentiment Analysis

**Figure 1:**
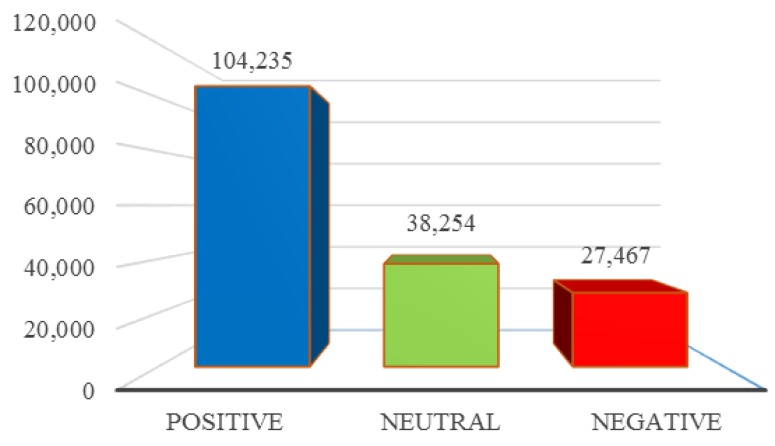
Polarity over the entire collection.

Figure 1 charts the number of positive, negative and neutral tweets over the entire collection. As readers may see, positive tweets—61% of the total—are more common than negative—16% of the total—or neutral ones—23% of the total.

Both commercial and academic researchers have proposed a number of metrics to estimate the overall sentiment expressed towards particular topics on social networks. A common metric for this purpose is the *net sentiment rate* (NSR) [50,51].

The (NSR) is defined as the subtraction of the number of negative conversations—negative tweets in our case—from the number of positive conversations—positive tweets—divided by the total number of conversations—total number of tweets. In other words,

(1) $$NSR=\frac{Positive\;tweets-Negative\;tweets}{Total\;number\;of\;tweets}$$

Table 7 displays the NSR for each of the hashtags and phrases considered in our study. Note that #lastchildinthewoods, #naturedeficitdisorder, #parkprescriptions and the phrase “nature deficit disorder” have negative NSRs. The hashtags #parkprescriptions and #naturedeficitdisorder have the lowest NSR in the collection, while #outdoors and #natureheals have the most positive NSRs. We explain below our interpretation of these findings.

**Table 7:** Net sentiment rate (NSR) per hashtag.

NDD Hashtags/Phrases		Tweets Retrieved
#lastchildinthewoods		−0.14
#nochildleftindoors		0.67
#leavenochildinside		0.40
#naturedeficitdisorder		−0.89
#thenatureprinciple		-
#vitamin N		0.19
nature deficit disorder		−0.62
**Ndd-Related Hashtags**		**Tweets Retrieved**
#getoutside		0.49
#goplay		0.43
#outdoorfamilies		0.64
#playoutside		0.43
#qualityfamilytimeinnature		-
**Nature–Health Hashtags**		**Tweets Retrieved**
#naturecures		0.64
#natureheals		0.72
#parkprescriptions		−0.95
**Generic-Nature Hashtags**		**Tweets Retrieved**
#green		0.52
#outdoors		0.77
#wildlife		0.38

**Figure 2:**
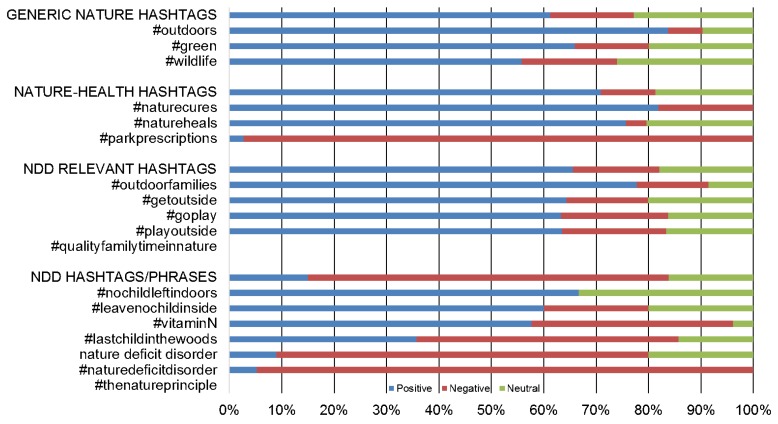
Tweet polarity per hashtag/phrase.

Figure 2 displays stacked histograms to visualize the polarity per hashtag—the vertical axis displays the different hashtags, phrases, and categories involved in the study, and the horizontal axis presents the percentage of tweets that are positive, negative and neutral divided by category.

We propose that the negative quality of the NSR for the hashtag #naturedeficitdisorder and the phrase “nature deficit disorder” is caused by the presence of comments in tweets that refer to negative behaviors and afflictions commonly associated with the causes and consequences of *NDD*, rather than opinions about *NDD* itself. Consider the following tweets:

@Sam10k Too many parents glued to #Electronic devices setting bad example! #NatureDeficitDisorder http://t.co/eivBfx1gmY

Link to childhood #depression #ADHD and #obesity “Nature Deficit Disorder”. Less screen time & more green time http://t.co/zdtdKTYlTe

The first tweet and its attached link are intended to critically evaluate families who seem to be getting worse at communicating—presumably, due to parents failing to set a good example to their children. The author of this tweet highlights a behavior that she strongly disapproves. As a result, AlchemyAPI classifies the text as negative—indeed, the score is −0.687712. Similarly, the second tweet—whose score is −0.74948—associates *NDD* with conditions such as depression, attention deficit hyperactivity disorder (ADHD) and obesity. *NDD* is not negatively qualified within this tweet, but its associations are.

In contrast to sentiments for the hashtag #naturedeficitdisorder and the phrase “nature deficit disorder”, the hashtag #nochildleftindoors comprises only three tweets, but all of them are associated with positive behaviors—in the view of their authors—and in some cases a cheerful, almost celebratory, phrasing style. Table 8 lists the tweets included in the hashtag #nochildleftindoors.

**Table 8:** Tweets comprised by the hashtag #nochildleftindoors.

Tweet	Polarity	Score
A Lake George Conservancy program is trying to foster stewardship in the next generation. #nochildleftindoors http://t.co/n9C4s4PEtc	positive	0.0531282
Watching my son go crazy on his #firstmackerel #familyfunday #nochildleftindoors #takeyourkidsfishing… http://t.co/z2LqQnw5FT	neutral	0.0505909
Best day on the water is one spent with my boys! #familyfunday #nochildleftindoors #boating #sandiego… http://t.co/XScgRxyNMx	positive	0.466531

Similar kinds of tweets can be found under the hashtags #natureheals and #outdoors, which have the most positive NSRs. The case of the hashtag #parkprescriptions is slightly different though: #parkprescriptions is made up of a small number of tweets published during the length of our study—37 in total. However, 36 of these tweets refer to the following post, which AlchemyAPI classifies as negative with a score of −0.208067.

#ParkPrescriptions: New Treatment for #Obesity #GLV http://t.co/2LJyaUwB5e

We contend that the post above, and consequently the 36 tweets that included it, could have been considered neutral, as the post is simply reporting on a new treatment, rather than criticizing or disqualifying it. The automatic assessment of polarity made by AlchemyAPI is not flawless and, in this case, it does have an adverse impact on the computation of the NSR. Had the post in question been marked as neutral, the NSR for #parkprescriptions would have been 0.03, rather than −0.95.

### 3.2. Time Course of Tweets

**Figure 3:**
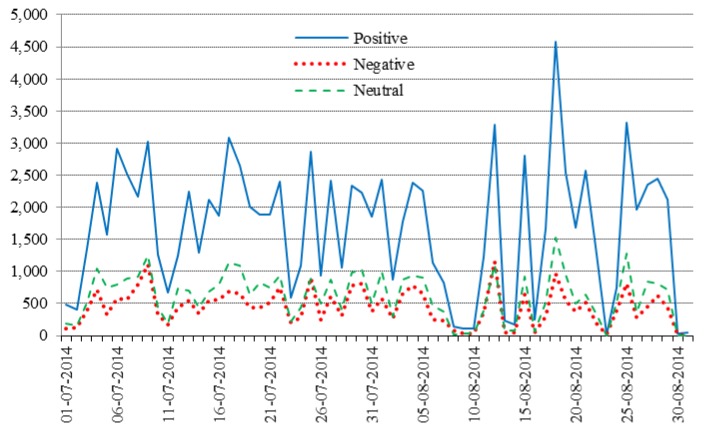
Tweet polarity on a day-per-day basis.

Figure 3 shows the number of positive, negative and neutral tweets published on a day-by-day basis over the two-month length of the study. As readers may see, the sentiment expressed on the tweets was primarily positive on every single day of the experiment. Additionally, neutral tweets were published more often than negative ones, with the exception of a small number of days, such as 25 July 2014 and 12 August 2014, when there were slightly more negative tweets than neutral ones.

It is worth noting that we captured a particularly large number of tweets on 9 July 2014—specifically, we captured 5373 tweets in total on 9 July 2014, and 3023 of them were positive. A semi-final match of the 2014 FIFA World Cup took place exactly on that day, and this might be the reason why a high Twitter traffic was observed on 9 July 2014 (Twitter reported a strong growth in 2014 driven by the “heavy” use of the service made by soccer fans around the world during the World Cup tournament, which spanned June and July 2014 [52]. Indeed, some of the tweets in our collection do make reference to the World Cup; yet, the number of such tweets is too small to think that they caused a significant burst in our statistics—the total number of tweets comprising the words “World Cup” or the hashtag #WorldCup in our collection is 107, and only 13 of them were published on 9 July 2014. Still, the fact that a larger number of users were active on the day of the semi-final match might have contributed to our gathering of a greater quantity of tweets. For illustration purposes, Table 9 exhibits some examples of the World Cup tweets that we retrieved. We have highlighted in bold font the hashtags that are associated with our study, and the words or hashtags referring to the 2014 FIFA World Cup.

The largest number of tweets in our collection was captured during the period 12–25 August 2014, which coincides with the time when the disputed circumstances of the shooting of Michael Brown, in Ferguson, Missouri, US—a suburb of St. Louis—and the subsequent protests and civil unrest received considerable attention in Twitter—both in the US and abroad. There were more than 18 million Tweets labeled with the hashtag #Ferguson in August 2014 [53]. Again, the fact that a larger number of users were active during the aftermath of this event might have contributed to our retrieving of a greater quantity of tweets.

**Table 9:** World Cup tweets published on 9 July 2014.

Tweet	Date + Time
Are you making the most of this amazing weather? #sunsout #outdoors #exercise #sports #tennis #Wimbledon #golf #open #football #WorldCup #AH	09-07-2014 10:11:12
How green is the 2014 World Cup?: This year’s tournament just brought a world of pain to Brazilian socc... http://t.co/F5z2ytYTve #green	09-07-2014 11:22:45
Congrats #Germany #worldcup! Freiburg, a #Green City! 660Ha of green spaces, bicycle trails, solar-architecture. Visit @freiburg	09-07-2014 16:05:12

### 3.3. Retweet Analysis

There were 78,531 retweets in the collection—*i.e.*, there were 78,531 posts in our collection (45% of the total number of posts) whose content republished original material posted within the length of our experiment.

**Figure 4:**
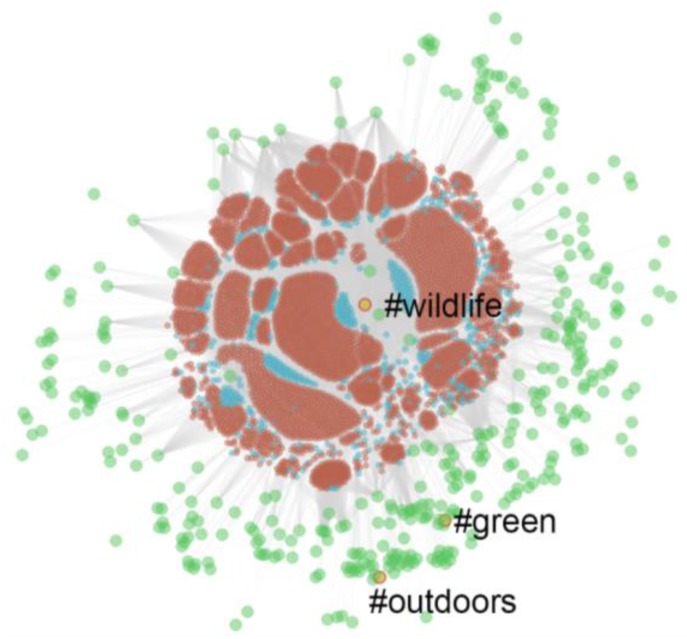
Hashtags appearing in tweets that were retweeted more than 5000 times.

Figure 4 shows a *force-directed* graph representing the tweets and retweets that we retrieved. The blue sections in Figure 4 represent the tweets that we retrieved, the brown sections represent the retweets that we retrieved, and the green circles represent the hashtags associated with them. We use a force-directed graph to visualize our data in a two-dimensional space, where the edges—which are drawn in gray every time a hashtag is included in a retweet—have more or less equal length, and there are as few crossing edges as possible.

We employed a bespoke software platform designed at *Robert Gordon University* to draw Figure 4—such a platform was adapted to deal with large volumes of tweets. We loaded up our collection of retweets into a *Neo4j* datastore—Neo4j is a popular open-source graph database (Neo4j^®^—Neo Technology, Inc. [54]). The advantage of this approach is that we can store our hashtags, tweets and retweets in the form of “nodes”, and the inclusion relationship between hashtags and retweets in the form of “edges”. For simplicity, we only worked with tweets that were retweeted more than 5000 times during the length of the study—thus, a hashtag such as #getoutside does not appear in Figure 4, because it was mentioned in more than 5000 tweets but none of those tweets was retweeted more than 5000 times. Figure 4 also shows that the retweets in our study refer predominantly to the hashtag #wildlife—hence, the tweets in our graph were clustered around this major hashtag.

**Figure 5:**
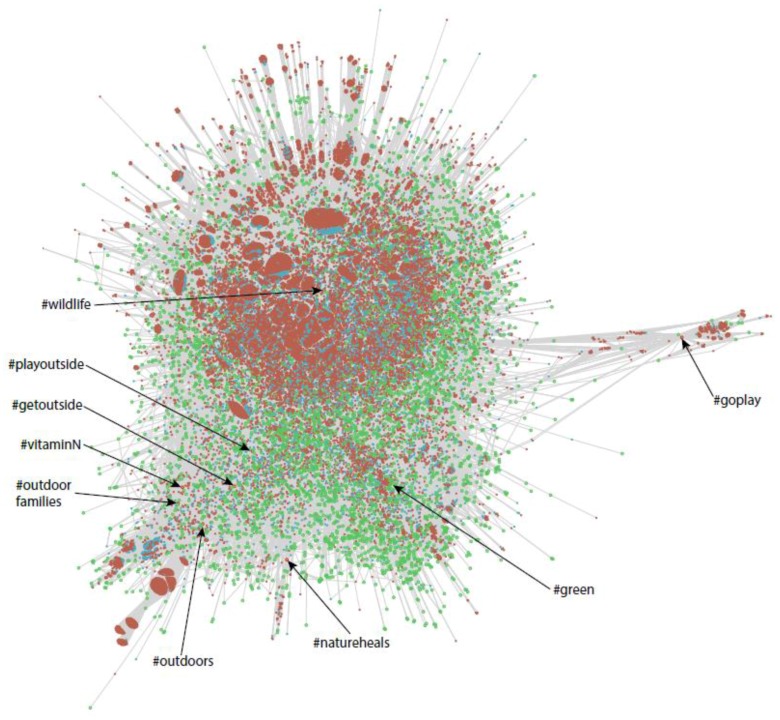
Tweets, retweets and hashtags distribution (100,000 sample).

To further analyze the distribution of tweets, retweets and hashtags, and to explore possible linkages between hashtags, we created Figure 5. Figure 5 renders a sample of 100,000 tweets that comprised more than one hashtag and all the hashtags that are mentioned more than once—recall that the blue sections represent the tweets that we retrieved, the brown sections represent the retweets that we retrieved, and the green circles represent the hashtags. Figure 5 shows the dominance of the #wildlife hashtag and how the hashtags chosen for our study covered separate sub collections of tweets that are quite divergent. For example, the other generic nature hashtags, #green and #outdoors do not appear to overlap much with #wildlife. *NDD*-related hashtags are also widespread—*i.e.*, #goplay is quite separate from the bulk of the graph. However, #getoutdoors and the *NDD* hashtag, #vitaminN, are relatively closely aligned with the more general concept #outdoors. Thus, our choice of hashtags actually allowed us to cover a large, heterogeneous range of tweets.

**Figure 6:**
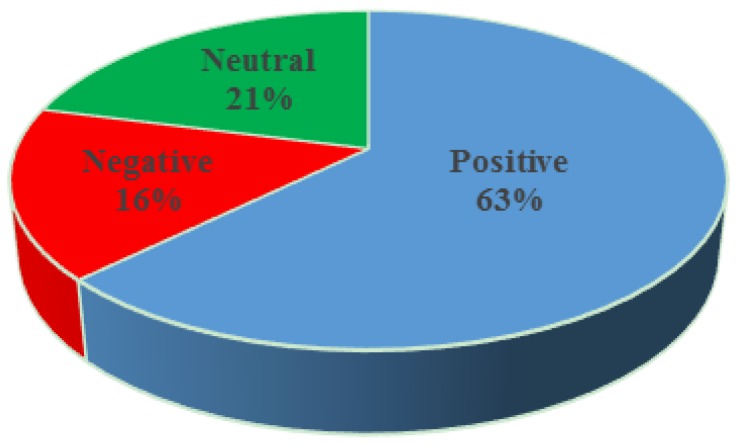
Retweet polarity.

The polarity of retweets is rather similar to the polarity of the collection as a whole. Figure 6 shows that 63% of the retweets in our collection are positive, 21% are neutral and only 16% are negative, which are very similar numbers to those that described the polarity of the entire collection. Recent research on word-of-mouth spread in Twitter [55] suggests that tweets with positive sentiment spread 15%–20% more than tweets containing a negative sentiment. Therefore, we can expect that our collection of original, primarily positive, tweets was likely to be retweeted further and for longer than a negative collection.

### 3.4. Message Themes

As a step in finalizing our dataset for an in-depth qualitative analysis that looks at themes, we quantified the other hashtags included in our identified set of tweets. Table 10 displays the top ten hashtags that were recorded most often as part of the group of tweets in each of our categories. For each hashtag in Table 10, we indicate its frequency of appearance. It should be observed that we only monitored 17 hashtags and one phrase, as stated in Section 2.1—the hashtags that we monitored are highlighted in bold font in Table 10. However, the tweets that we captured comprised other hashtags in addition to those that we were monitoring.

We expected in advance that certain hashtags such as #wildlife, #green, #outdoors and #getoutside would be in this list of most frequent hashtags, because we retrieved a large number of tweets using them. Perhaps not surprisingly, the only hashtag to appear in the top ten in all four of our subgroups was #nature.

*NDD* hashtags include several about #physicalactivity, which is consistent with the outdoors as a preferred setting for physical activity. An alarming group of hashtags include #beheadingchristians, #isis, #nukeisis. On further investigation, we found that all these hashtags were used in a tweet along with #vitaminN that had a very different meaning than we would expect of “N” for “Nature”. Such a tweet appears below,

#ISIS needs some #VitaminN #NukeISIS #StopISIS #BeheadingChristians #Iraq #Yazidis #Sinjar #StandWithIsrael #tcot http://t.co/KjuFhqxWU2

In the case of the tweet shown above, “N” stands for “Nuke” or “Nuclear”. The 17 instances of the hashtags #beheadingchristians, #isis, #nukeisis include the original tweet and its retweets. This finding suggests that looking at the most frequent hashtags in a group of tweets can surface content that needs to be excluded as non-relevant to the desired inquiry.

The *NDD*-related hashtags are topped by our preselected hashtags: #getoutside, #goplay, #outdoorfamilies, #playoutside. Other hashtags, #nature, #hiking, #summer, #outdoors—one of our generic nature hashtags—also make a strong appearance. Taken together, these suggest active outdoor play, which is a strong concept in Louv’s work [12]. The anomalous hashtags appearing here are #adda52rummy and #rummy. These refer to the popular online *rummy game* from India that runs on an app that can be taken anywhere, including the great outdoors. Again, looking at the hashtags allows us to quickly target content that might be appropriate to exclude from the analysis.

The Nature–health hashtags have a very different feel to them from the previous two categories, one that may be tapping the spiritual transcendence that many people experience in nature [56]: #church, #earthtemple, #cosmicconsciousness, #iampeace, #nows—the momentary present. #glv may stand for *Greater London Volunteering* (Greater London Volunteering|London's leading voice for volunteering: [57]) which supports many nature-based volunteer opportunities. And #obesity is a major global health crisis that nature activity may help ameliorate in important ways.

Finally, in the Generic-nature hashtags, we find classic nature content, such as #animals, #birds, commonly related activities like #photography, #art, and place-based sentiment like #love and #beautiful.

Our next level of content overview included determining the most frequently used words within the tweets. In accordance with information retrieval practices, we removed the *stop-words*—*i.e.*, extremely common words that are of little value in helping identify characteristic themes—from the tweets, prior to counting word occurrences. The stop-word list that we used was built by Salton and Buckley for the *SMART* information retrieval system [58], and it guaranteed that semantically non-selective words—such as articles, pronouns and prepositions—were deleted from the occurrences count.

Table 11 displays the 10 most common words in each of our categories. Notice that two of the top three words in *NDD* hashtags could be interpreted as negative words: *disorder* and *deficit*. These words likely contribute to the negative sentiment analysis for this category. The other categories are largely made up of positive words, descriptive of the positive experiences in nature.

**Table 10:** Most frequent hashtags per category.

**NDD Hashtags/Phrases**	
**Hashtag**	**Frequency**
#nature	88
**#naturedeficitdisorder**	**61**
**#vitaminN**	**52**
#physicalactivity	28
#beheadingchristians	17
#iraq	17
#isis	17
#nukeisis	17
#sinjar	17
#standwithisrael	17
**NDD-Related Hashtags**	
**Hashtag**	**Frequency**
**#getoutside**	**6100**
**#goplay**	**1593**
**#outdoorfamilies**	**1050**
**#playoutside**	**692**
#nature	438
#adda52rummy	288
#hiking	279
#rummy	245
#summer	239
**#outdoors**	**193**
**Nature–Health Hashtags**	
**Hashtag**	**Frequency**
**#natureheals**	**475**
#nature	119
#church	91
#earthtemple	67
#cosmicconsciousness	64
#iampeace	59
**#parkprescriptions**	**37**
#glv	36
#obesity	36
#nows	35
**Generic-Nature Hashtags**	
**Hashtag**	**Frequency**
**#wildlife**	**110,430**
**#green**	**32,589**
#nature	25,790
**#outdoors**	**20,400**
#photography	17,427
#animals	14,109
#birds	4989
#love	3970
#art	3331
#beautiful	3267

**Table 11:** Most frequent words contained in the tweets.

**NDD Hashtags/Phrases**	
**Hashtag**	**Frequency**
Disorder	331
Nature	314
Deficit	263
Children	106
Child	103
Kids	94
Woods	70
Saving	62
Suffer	53
Read	45
**NDD-Related Hashtags**	
**Hashtag**	**Frequency**
Day	621
Play	474
Today	471
Kids	435
Great	406
Time	389
Weekend	363
Summer	302
Beautiful	301
Park	291
**Nature–Health Hashtags**	
**Hashtag**	**Frequency**
Walk	95
Equivalent	93
Considered	91
Father	91
Love	75
Soul	58
Silence	57
Waiting	57
Wake	57
Nature	44
**Generic-Nature Hashtags**	
**Hashtag**	**Frequency**
Animal	7987
Photography	7024
Day	4843
Great	4209
Wildlife	3757
World	3571
Photo	3473
Find	3236
Love	3189
Sign	3159

Further mapping of these common words as well as thematic analysis of complete tweets is beyond the scope of this paper, but has been fruitful in other public health contexts including dental pain surveillance [59], surveillance of the dissemination of information around H1N1 during an outbreak [60] and analysis of misunderstandings about and the misuse of antibiotics [61].

### 3.5. Tweet Originators

The 176,494 tweets considered in the study were published by 74,485 different users, for an average of 2–3 tweets per user; however, some users post much more frequently. Table 12 shows the number of tweets published by the 15 users with the largest presence in our collection, accounting for 6% of all tweets. Table 12 also shows, for each user, the total number of *NDD* relevant tweets—we refer to *NDD* relevant tweets as those that contain at least one hashtag or phrase included in our study—and the total number of tweets published at the time of writing, regardless of their connection to *NDD*, and the corresponding number of followers listed on their profile at the time of writing, as an indicator of how influential they might be.

**Table 12:** Most publishing users.

User ID	*NDD* Relevant Tweets	Total Tweets (Thousand)	Followers (Thousand)
@pinkbigmac	3443	607	8.2
@PhuketDailyNews	2470	582	12
@PHOTOSintheWILD	1239	88	12
@chaebae	967	359	13
@ecOikoinfo	834	35	2.1
@environsecnews	665	96	0.6
@bowhuntingAddic	606	53	0.2
@Golf_And_Hunt	594	53	0.1
@LetsGoForAHike	592	22	0.3
@NappeeMatthieu	510	-	-
@ImVarghese	492	293	2.5
@WhyWeClimb	474	24	1.4
@FredHolmesPhoto	461	18	0.2
@africam	446	23	13
@SipoArt	428	290	23

Some of the most frequent publishers in the collection appear to be news syndication services—like, @PhuketDailyNews and @environsecnews, which publish material gathered from the *Sub Saharan African Concise News Service*. These services are largely produced by automatic aggregation. The most prolific user in our collection is @pinkbigmac—a service that allows the virtual exploration of travel destinations around the world. However, @pinkbigmac is not particularly influential, since it is not followed by a large number of users. It is notable that two names of top publishers indicate a link with the hashtag #photography: @photosinthewild and @FredHolmesPhoto—“photography” is also a common word found in our identified set of tweets. In this context, *Sipo Liimatainen* (@SipoArt), a surrealist and abstract Scandinavian artist, is the most influential user—followed by more than 23,000 users.

### 3.6. Discussion

We set out to discover if a social media channel like Twitter could provide useful data about the public viewpoint and the social dissemination of the concept *nature-deficit disorder.* In doing so we have described our methods of data gathering, applied an emerging methodology, sentiment analysis, and mapped the hashtag, tweets and retweets related to content about nature and health. Sentiment analysis has previously been used to track opinion about health care reforms over time [62]. Another study examined the use of Twitter in the dissemination of ideas about antibiotic use, using traditional methods to manually code the information in tweets [61]. We employed more machine-based approaches and attempted to identify issues and challenges associated with these methods and the desire to capitalize on the “big data” available.

The dissemination of messages relating to *NDD*, such as going outside for play or other activities that afford a greater connection with nature, is important for the uptake of healthier behavior and improvement of health and wellbeing. Previous studies have shown that the negative framing of messages has a lower effect on attitudes, intentions and behavior than messages framed positively [63]. Our results above suggest that some hashtags are more commonly associated with negative sentiments—suggesting a negative framing of the tweet in question. This may affect the impact of the tweet on the reader. Tweets with the #naturedeficitdisorder hashtag have been associated with predominantly negative sentiments—whereas other associated hashtags such as #natureheals and #getoutdoors have been associated with more positive sentiments. The uptake of the positive messages is also shown in the retweet polarity—tweets with positive sentiment are more likely to be retweeted [55]. There is increasing understanding of the need for the use of social media and other Web 2.0 strategies in disseminating public health messages, but strategic planning is needed to ensure messages are appropriately disseminated [64,65]. By examining the way different hashtags are related and the sentiments associated with them, health practitioners may be able to improve their dissemination strategies.

The results may in part be affected by the fact that in its name *NDD* is framed in a negative way. Tweets including the words “deficit” or “disorder” could be considered by automatic systems as being more negative in sentiment. However, this does suggest that public health officials and organizations interested in promoting outdoor exposure to reduce the potential negative health impacts might consider using more positive language in communications. For example, the wider use of the hashtags #getoutside or #outdoorfamilies in tweets from such groups may influence the response of Twitter users to the concept and aid in the wider dissemination of the ideas.

We have documented how the general Twitter traffic and sentiment in our dataset swells and ebbs over time; yet, it is consistently relatively positive about nature and health-related concepts. This is compatible with other research demonstrating positive emotions elicited in nature [66,67,68]. Force directed graphs were useful in showing the relationships between tweets and retweets—certain hashtags are shown to inhabit a different “space” in Twitter. The examination of hashtags that occur most frequently suggest that tweets with a nature–health hashtag may be more likely to contain elements that are suggestive of the spiritual transcendence that many people experience in nature [56]. Analysis of frequently occurring hashtags may also assist in the cleaning of datasets.

We have demonstrated the process of preparing a Twitter dataset that can expand our understanding of *NDD* and related nature–health ideas. Ironically, the very technology that might lead to *NDD* may be part of the way to understand and communicate deeply held feelings towards nature and how nature experiences affect human health and wellbeing.

### 3.7. Limitations

In conducting this study using the new methods of Twitter information-mining, sentiment analysis, and mapping of retweets, we encountered a number of important limitations. First, we were limited in the number of tweets that could be collected with each query, meaning that some very prominently used hashtags—e.g., #wildlife, #green—contained truncated data. This could bias our results. However, we collected over 175,000 tweets and as such have a relatively robust sample. Second, we used a limited set of hashtags based on expert opinion. Had we used an iterative process whereby little-used hashtags were dropped and commonly identified hashtags were added, we might have obtained a richer dataset.

While quantifying sentiment, we recognized that the results are based on the assigned emotional polarity of words in the software’s dictionary. *AlchemyAPI* does not publish its dictionary and assignment, so while we can deduce the categorization of some words, in general this is opaque. Recent work also shows little agreement in sentiment analysis conducted with different software applications that may rest on this lack of consensus in assigning emotional valence [69].

Another caveat to successful use of social media data has to do with the timing of data collection. We did not link our two-month data collection to a specific event or public campaign about *NDD*, so the available data was, in fact, sparse, merely quantifying the persistent background discourse. We also saw how world events, such as the FIFA World Cup and the Ferguson killing in the US, may influence the day-to-day volume of available tweets, even on an unrelated topic [70]. Seasonality may also play a role—and previous analysis has shown that the sentiment of tweets is related to the weather—with rainfall and snow depth having been shown to be significantly linked to increased negative mood [71].

We also found that while mapping tweets, retweets and hashtags is possible, detailed qualitative analysis of the statements in tweets will be challenging, given the brevity of the content and the extraordinary high volume of tweets available for analysis. Analyzing selected tweet subsets and using mind mapping tools along with qualitative methods software may assist in illuminating diverse themes and their relationships [21,72]. Irvine and Warber [68] have previously shown the utility of content analysis of brief responses of park users to broaden our understanding of their motivations and perceived benefits associated with being in a park. The qualitative analysis of complete tweets in this dataset could be a fruitful source for understanding what nature means to a group of people, the technologically able, that might not be tapped in other sorts of studies.

Finally, we also encountered the challenge of identifying the originators of tweets. It is appealing to think of them as individuals, tweeting from their cell phones in pristine environments, or sending photos of exquisite natural beauty to the public at large, but our look at influential users revealed automated news syndication services, professional artists and photographers, and travel facilitators, among others. An important future step in the analysis of this dataset would be to parse the sample into individual users *vs.* commercial or non-profit organizations, in order to better understand their divergent opinions.

## 4. Conclusions

With the growing interest in how online sedentary activity might hinder the contact of both children and adults with the outdoors and offline healthy practices, we have presented an exploration of social media activity related to human health and the environment. *Nature-deficit disorder* is not yet regarded as a medical condition—it is not recognized by any medical coding schemes, such as ICD-10 [73], or the DSM-5 [74], the American Psychiatric Association’s classification and diagnostic tool. However, evidence collected in various countries suggests that the rates of obesity, self-harm and mental health disorders have climbed significantly [75], while people are spending less time outdoors [13] and sedentary activity becomes prevalent [76]. Numerous studies have considered various health and behavioral issues associated with this, as well as environmental considerations [76].

Social Awareness Streams, like those found in Twitter, have implications for public health promotion efforts [77,78]. Unique features of social media, such as mass customization, interactivity and convenience are beneficial to health communication and promotion efforts [79], and convert what would have been “private” health entries in a journal into interactive “public” disclosures and potential points of discussion among contacts or followers.

Analysis of Twitter data using hashtags relevant to the concept of *NDD* suggests there are significant differences in the way that messages associated to certain hashtags are framed. This may influence the uptake and wider dissemination of these messages via retweeting. It is important that public health officials and those seeking to disseminate knowledge regarding *NDD* consider carefully both the hashtags used and the sentiment of the message.

The results presented here are based on a limited sample of Twitter data. Further use of sentiment analysis in the assessment of Twitter data regarding emerging environmental health concepts may aid in better understanding the way such concepts are being used by the public and organizations. This in turn may lead to improvements in the use of Twitter for the communication of public health messages.

## References

[1] Hartig T., Mitchell R., de Vries S., Frumkin H. (2014). Nature and Health. Annu. Rev. Public Health.

[2] Kaplan S., Berman M.G. (2010). Directed attention as a common resource for executive functioning and self-regulation. Perspect. Psychol. Sci..

[3] Ward Thompson C., Aspinall P., Roe J. (2014). Access to green space in disadvantaged urban communities: Evidence of salutogenic effects based on biomarker and self-report measures of wellbeing. Proc. Soc. Behav. Sci..

[4] Maas J., Verheij R., de Vries S., Spreeuwenberg P., Schellevis F., Groenewegen P. (2009). Morbidity is related to a green living environment. J. Epidemiol. Community Health.

[5] Thompson Coon J., Boddy K., Stein K., Whear R., Barton J.L., Depledge M.H. (2011). Does participating in physical activity in outdoor natural environments have a greater effect on physical and mental wellbeing than physical activity indoors? A systematic review. Environ. Sci. Technol..

[6] Marselle M.R., Irvine K.N., Warber S.L. (2014). Examining group walks in nature and multiple aspects of well-being: A large-scale study. Ecopsychology.

[7] Barton J., Pretty J. (2010). What is the best dose of nature and green exercise for improving mental health? A multi-study analysis. Environ. Sci. Technol..

[8] Stamatakis E., Coombs N., Jago R., Gama A., Mourão I. (2013). Associations between indicators of screen time and adiposity indices in Portuguese children. Prev. Med..

[9] Liu G.C., Wilson J.S., Qi R., Ying J. (2007). Green neighborhoods, food retail and childhood overweight: Differences by population density. Am. J. Health Promot..

[10] Vaughan A. (2013). Four Out of Five UK Children “Not Connected to Nature”. http://www.theguardian.com/environment/2013/oct/16/uk-children-not-connected-nature-rspb.

[11] Babey S.H., Wolstein J., Krumholz S., Robertson B., Diamant A.L. Physical Activity, Park Access and Park Use among California Adolescents. http://healthpolicy.ucla.edu/publications/Documents/PDF/parkaccesspb-mar2013.pdf.

[12] Louv R. (2005). Last Child in the Woods: Saving Our Children from Nature-Deficit Disorder.

[13] Moss S. Natural Childhood: A Report by the National Trust on Nature Deficit Disorder. http://www.lotc.org.uk/natural-childhood-a-report-by-the-national-trust-on-nature-deficit-disorder/.

[14] Warber S.L., DeHudy A.A., Bialko M.F., Marselle M.M., Irvine K.N. (2015). Addressing nature-deficit disorder: A mixed methods pilot study of young adults attending a wilderness camp. Evid. Based Complement. Altern. Med..

[15] Driessnack M. (2009). Children and nature-deficit disorder. J. Spec. Pediatr. Nurs..

[16] Brown P. (1995). Naming and framing: The social construction of diagnosis and illness. J. Health Soc. Behav..

[17] Finfgeld-Connett D. (2015). Twitter and health science research. West. J. Nurs. Res..

[18] Charles-Smith L.E., Reynolds T.L., Cameron M.A., Conway M., Lau E.H.Y., Olsen J.M., Pavlin J.A., Shigematsu M., Streichert L.C., Studa K.J. (2015). Using social media for actionable disease surveillance and outbreak management: A systematic literature review. PLoS ONE.

[19] Lister C., Royne M., Payne H.E., Cannon B., Hanson C., Barnes M. (2015). The laugh model: Reframing and rebranding public health through social media. Am. J. Public Health.

[20] Woo H., Cho Y., Shim E., Lee K., Song G. (2015). Public trauma after the Sewol Ferry disaster: The role of social media in understanding the public mood. Int. J. Environ. Res. Public Health.

[21] Hays R., Daker-White G. (2015). The care data consensus? A qualitative analysis of opinions expressed on Twitter. BMC Public Health.

[22] Balahur A., Mihalcea R., Montoyo A. (2014). Computational approaches to subjectivity and sentiment analysis: Present and envisaged methods and applications. Comput. Speech Lang..

[23] Liu B. (2012). Sentiment analysis and opinion mining. Synth. Lectures Hum. Lang. Technol..

[24] Ji X., Chun S., Wei Z., Geller J. (2015). Twitter sentiment classification for measuring public health concerns. Soc. Netw. Anal. Min..

[25] Castellanos M., Dayal U., Hsu M., Ghosh R., Dekhil M., Lu Y., Zhang L., Schreiman M. (2011). LCI: A Social Channel Analysis Platform for Live Customer Intelligence.

[26] Kendall L., Hartzler A., Klasnja P., Pratt W. (2011). Descriptive Analysis of Physical Activity Conversations on Twitter.

[27] Naaman M., Boase J., Lai C.H. (2010). Is It Really about Me? Message Content in Social Awareness Streams.

[28] Twitter (2015). Welcome to Twitter. https://twitter.com/.

[29] Twitter Getting started with Twitter. https://support.twitter.com/articles/215585#.

[30] Twitter Using Hashtags on Twitter. https://support.twitter.com/articles/49309#.

[31] Twitter—Platform Engineering New Tweets Per Second Record, and How!. https://blog.twitter.com/2013/new-tweets-per-second-record-and-how.

[32] Twitter4J Twitter4J—Introduction. http://twitter4j.org/en/index.html.

[33] Twitter (2014). The Search API. https://twitter.com/search-home.

[34] Gonçalves P., Araújo M., Benevenuto F., Cha M. (2013). Comparing and Combining Sentiment Analysis Methods.

[35] Go A., Bhayani R., Huang L. (2009). Twitter sentiment classification using distant supervision. Processing.

[36] Read J. (2005). Using Emoticons to Reduce Dependency in Machine Learning Techniques for Sentiment Classification.

[37] Pak A., Paroubek P. (2010). Twitter as a corpus for sentiment analysis and opinion mining. LREC.

[38] Whissell C., Plutchik R., Kellerman H. (1989). The dictionary of affect in language. Emotion: Theory, Research and Experience.

[39] Zhang L., Ghosh R., Dekhil M., Hsu M., Liu B. Combining Lexicon-Based and Learning-Based Methods for Twitter Sentiment Analysis. http://www.hpl.hp.com/techreports/2011/HPL-2011-89.pdf.

[40] AlchemyAPI, Inc. AlchemyAPI. http://www.alchemyapi.com/.

[41] Sentiment Analysis with AlchemyAPI: A Hybrid Approach. http://resources.alchemyapi.com/white-papers/sentiment-analysis-a-hybrid-approach.

[42] Stankiewicz J. (2015). IBM Acquires AlchemyAPI, Enhancing Watson’s Deep Learning Capabilities. https://www-03.ibm.com/press/us/en/pressrelease/46205.wss.

[43] Meehan K., Lunney T., Curran K., McCaughey A. (2013). Context-aware intelligent recommendation system for tourism. IEEE.

[44] Rizzo G., Troncy R. Nerd: Evaluating Named Entity Recognition Tools in the Web of Data. http://porto.polito.it/2440793/1/wekex2011_submission_6.

[45] Saif H., He Y., Alani H. (2012). Semantic Sentiment Analysis of Twitter.

[46] Zemanta Trusted Content Discovery. http://blog.zemanta.com/.

[47] Thomson Reuters Open Calais™. http://www.opencalais.com/.

[48] Extractiv Extractiv Software. http://extractiv.com/.

[49] GitHub, Inc. DBpedia Spotlight. https://github.com/dbpedia-spotlight/dbpedia-spotlight.

[50] Takeuchi T. (2014). Analyzing Twitter with MATLAB. https://github.com/toshiakit/AnalyzeTwitter.

[51] Marsden P. (2010). Social Media Metrics—SIM Score *vs.* Net Reputation Score (NRS). http://digitalintelligencetoday.com/social-media-metrics-sim-score-vs-net-reputation-score-nrs/.

[52] Goel V. World Cup Gave Twitter a Big Burst in Traffic. http://www.nytimes.com/2014/07/30/technology/twitter-quarterly-earnings.html.

[53] Stricker G. The 2014 #YearOnTwitter. https://blog.twitter.com/2014/the-2014-yearontwitter.

[54] Neo Technology, Inc. Neo4j. http://neo4j.com/.

[55] Bornfeld B., Rafaeli S., Raban D.R. Electronic Word-of-Mouth Spread in Twitter as a Function of Message Sentiment. gsb.haifa.ac.il/~sheizaf/BorenfeldRafaeliRaban2014EOMMessageSentiment.pdf.

[56] Williams K., Harvey D. (2001). Transcendent experience in forest environments. J. Environ. Psychol..

[57] Greater London Volunteering—London’s Leading Voice for Volunteering. http://greaterlondonvolunteering.org.uk/.

[58] Salton G. (1990). Full text information processing using the SMART system. IEEE Data Eng. Bull..

[59] Heavilin N., Gerbert B., Page J.E., Gibbs J.L. (2011). Public health surveillance of dental pain via twitter. J. Dent. Res..

[60] Chew C., Eisenbach G. (2010). Pandemics in the age of Twitter: Content analysis of Tweets during the H1N1 outbreak. PLoS ONE.

[61] Scanfield D., Scanfield V., Larsen E. (2010). Dissemination of health information through social networks: Twitter and antibiotics. Am. J. Infect. Control..

[62] King D., Ramirez-Cano D., Greaves F., Vlaev I., Beales S., Darzi A. (2013). Twitter and the health reforms in the English National Health Service. Health Policy.

[63] Gallagher K., Updegraff J. (2012). Health message framing effects on attitudes, intentions and behavior: A meta-analytic review. Ann. Behav. Med..

[64] Dooley J.A., Jones S.C., Iverson D. (2014). Using Web 2.0 for health promotion and social marketing efforts: Lessons learned from Web 2.0 experts. Health Market. Q..

[65] Wehner M., Chren M.M., Shive M., Resneck J., Pagoto S., Seidenberg A., Linos E. (2014). Twitter: An opportunity for public health campaigns. Lancet.

[66] Ulrich R.S., Altman I., Wohlwill J. (1983). Aesthetic and affective response to natural environment. Human Behavior and the Natural Environment.

[67] Capaldi C.A., Dopko R.L., Zelenski J.M. (2014). The relationship between nature connectedness and happiness: A meta-analysis. Front. Psychol..

[68] Irvine K.N., Warber S.L., Devine-Wright P., Gaston K.J. (2013). Understanding urban green space as a health resource: A qualitative comparison of visit motivation and derived effects among park users in Sheffield, UK. Int. J. Environ. Res. Public Health.

[69] Araújo M., Gonçalves P., Cha M., Benevenuto F. iFeel: A System that Compares and Combines Sentiment Analysis Methods. http://dl.acm.org/citation.cfm?id=2577013.

[70] Goel V. World Cup Gave Twitter a Big Burst in Traffic. http://www.nytimes.com/2014/07/30/technology/twitter-quarterly-earnings.html.

[71] Li J., Wang X., Hovy E. (2014). What a Nasty Day: Exploring Mood-Weather Relationship from Twitter.

[72] Cole-Lewis H., Varghese A., Sanders A., Schwarz M., Pugatch J., Augustson E. (2015). Assessing electronic cigarette-related Tweets for sentiment and content using supervised machine learning. J. Med. Internet Res..

[73] World Health Organization (2010). International Statistical Classification of Diseases and Related Health Problems (ICD-10).

[74] American Psychiatric Association (2013). Diagnostic and Statistical Manual of Mental Disorders: DSM-5, United States.

[75] Centers for Disease Control & Prevention (2012). U.S. Obesity Trends. http://www.cdc.gov/obesity/data/trends.html.

[76] Hill J.O., Peters J.C. (1998). Environmental contributions to the obesity epidemic. Science.

[77] Chou W.S., Hunt Y.M., Beckjord E.B., Moser R.P., Hesse B.W. (2009). Social media use in the United States: Implications for health communication. J. Med. Internet Res..

[78] Paul M., Dredze M. You are what you tweet: Analyzing Twitter for public health. Proceedings of the AAAI Conference on Weblogs and Social Media.

[79] Neuhauser L., Kreps G.L. (2003). Rethinking communication in the e-health era. J. Health Psychol..

